# Potentiating angiogenesis arrest in vivo via laser irradiation of peptide functionalised gold nanoparticles

**DOI:** 10.1186/s12951-017-0321-2

**Published:** 2017-11-21

**Authors:** Pedro Pedrosa, Amelie Heuer-Jungemann, Antonios G. Kanaras, Alexandra R. Fernandes, Pedro V. Baptista

**Affiliations:** 1 UCIBIO, Departamento de Ciências da Vida, Faculdade de Ciências e Tecnologia, Universidade NOVA de Lisboa, Caparica, Portugal; 2 Institute for Life Sciences, Physics and Astronomy, Faculty of Physical Sciences and Engineering, University of Southampton, Southampton, SO17 1BJ UK

**Keywords:** Gold nanoparticles, Angiogenesis, Green laser, CAM assay, VEGFR pathway, Phototherapy

## Abstract

**Background:**

Anti-angiogenic therapy has great potential for cancer therapy with several FDA approved formulations but there are considerable side effects upon the normal blood vessels that decrease the potential application of such therapeutics. Chicken chorioallantoic membrane (CAM) has been used as a model to study angiogenesis in vivo. Using a CAM model, it had been previously shown that spherical gold nanoparticles functionalised with an anti-angiogenic peptide can humper neo-angiogenesis.

**Results:**

Our results show that gold nanoparticles conjugated with an anti-angiogenic peptide can be combined with visible laser irradiation to enhance angiogenesis arrest in vivo. We show that a green laser coupled to gold nanoparticles can achieve high localized temperatures able to precisely cauterize blood vessels. This combined therapy acts via VEGFR pathway inhibition, leading to a fourfold reduction in *FLT*-*1* expression.

**Conclusions:**

The proposed phototherapy extends the use of visible lasers in clinics, combining it with chemotherapy to potentiate cancer treatment. This approach allows the reduction of dose of anti-angiogenic peptide, thus reducing possible side effects, while destroying blood vessels supply critical for tumour progression.

**Electronic supplementary material:**

The online version of this article (10.1186/s12951-017-0321-2) contains supplementary material, which is available to authorized users.

## Background

In cancer development, tumour growth is associated with increasing need for oxygen and nutrients to supply the uncontrolled multiplication of malignant cells. As such, tumours release a series of growth factors to stimulate the formation of new blood vessels—neo-angiogenesis, which in turn provide for the very needed nutrients [1, 2]. Among these, the vascular endothelial growth factor (VEGF) is responsible for the angiogenic trigger, leading to the foundation of new blood vessels surrounding the tumour tissue, thus allowing for its exponential growth [3, 4]. Upregulation of VEGF is a critical step in endothelial cell growth, survival and proliferation. The rapid growth of endothelial around tumours, results in an unorganised and leaky vasculature that potentiate further tumour growth and metastasis [3, 5]. Several FDA approved anti-angiogenic agents have already shown the potential to decrease tumour-induced neo-angiogenesis, but the considerable side effects towards the normal vascular architecture in other organs, and the development of resistance to these drugs decreases their effect [2, 6–8].

Nanoparticles hold great promise to deliver therapeutic agents selectively to tumours by increasing the local concentration of active molecules via the enhanced permeability and retention (EPR) effect at the tumour site [9–11]. EPR allows for the passive accumulation of the nanoparticles within the tumour site, where the confinement in a reduced volume permits lower dosages, improving the therapeutic effect while decreasing side effects [6, 10]. We have previously shown that gold nanoparticles (AuNPs) functionalised with an anti-angiogenic peptide targeting the neuropilin-1 receptor (NRP-1), promote receptor internalisation and efficiently arrest angiogenesis in vivo [12, 13]. NRP-1 is a transmembrane glycoprotein and a co-receptor for VEGF [4, 14]. Although the precise mechanism for VEGF binding to NRP-1 in angiogenesis and in endothelial function remains to be unveiled, pre-clinical studies have linked NRP-1 blockade to suppression of tumour growth by blocking angiogenesis [15, 16]. The effect of our peptide-functionalised nanoparticles on human umbilical vein endothelial cells (HUVEC) was potentiated by the simultaneous combination of laser irradiation in the near-infrared [17, 18]. However, precise irradiation on the target site is possible by using a laser of visible light (green). Indeed, green lasers have been used in medical surgery as a safe tool for tissue ablation without bleeding, inducing photocoagulation of capillaries and minimal destruction of surrounding tissue [19–21]. Initially used in retinoblastoma focal therapy, their applications were broadened to hyperthermia directed at epithelial cancers or photodynamic therapy, for accurate tumour removal or photo activation of chemotherapeutic agents [19–23]. AuNPs with a diameter < 20 nm show a very intense localised surface plasmon resonance (LSPR) band centred at 520 nm (close to the standard wavelength of surgical green lasers) that can be used for localised plasmon photothermal therapy. Spherical AuNPs coupled to visible lasers have been reported as an efficient tool for hyperthermia, with increased photothermal efficiency [24] and are less influenced by particle polydispersity compared to gold nanorods [25]. In addition, green lasers have an increased effect on blood vessels, being an active tool against angiogenic conglomerates around tumours.

Here, we propose the combination of green laser irradiation with administration of a nanoformulation of 13 nm spherical AuNPs functionalised with an anti-angiogenic peptide for the focalised phototherapy towards blockade of neovascularisation in vivo. For this, we used the chicken chorioallantoic membrane (CAM) model of angiogenesis to evaluate the anti-angiogenic effect of gold nanoconjugate formulations. The CAM is an extraembryonic membrane that supports a dense capillary network, extensively vascularised and easily accessible. It has been broadly used to test the efficacy and mechanisms of action of proangiogenic and antiangiogenic molecules in vivo [26, 27].

## Results and discussion

**Fig. 1:**
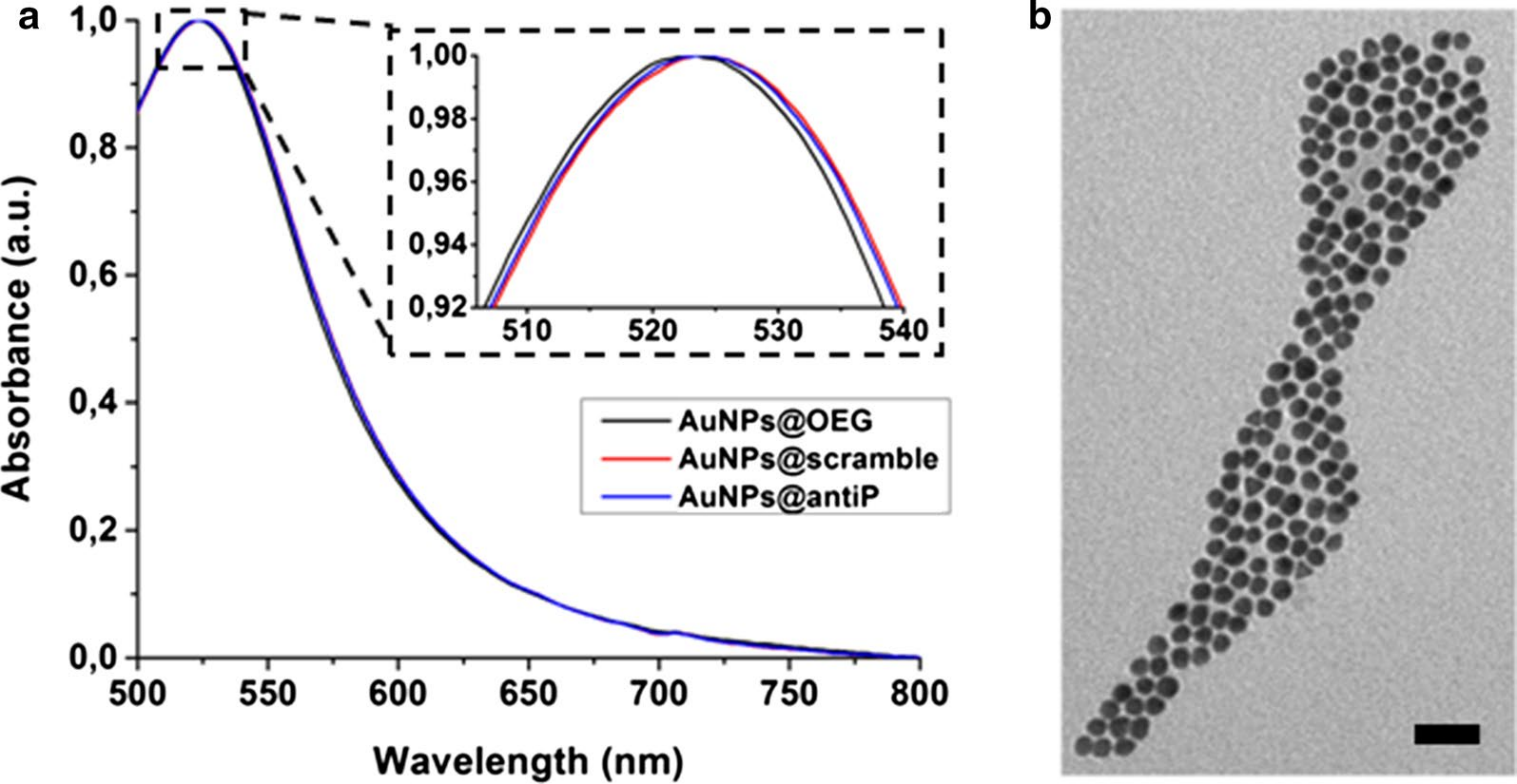
**a** Characterization of functionalized AuNPs by UV–Vis spectroscopy. A slight red shift in the UV–Vis spectrum of AuNPs@antiP/scramble infers the successful functionalisation with these peptides. **b** Transmission electron microscopy of citrate AuNPs. Scale bar is 50 nm

AuNPs with an average diameter of 13 ± 2 nm were synthesised via the citrate reduction method of Turkevich et al. [28], functionalised with oligo ethylene glycol (OEG) (AuNPs@OEG) to provide for increased stability in complex biologic media, and subsequently conjugated with an anti-angiogenic peptide—KATWLPPR, (AuNPs@antiP), capable to interact with NRP-1 receptor and inhibit the VEGF pathway [12]. As a control, a scramble peptide (KPRQPSLP) (AuNPs@scramble) was used. The nanoconjugates were characterised by UV–Vis and TEM, (Fig. 1, Additional file 1: Figure S1.1). Actinometry was performed at irradiation conditions to determine the exact amount of energy reaching the irradiation spots (Additional file 1: Figure S1.2).

Then, to understand the impact of irradiating AuNPs on the biological matrix (chicken egg), we irradiated the egg white for 5 min. at 3.83 W cm^−2^. Albumen proved to be a good model matrix since it is transparent to visible light and mostly composed of water and albumin, where the effect of irradiation combined with AuNPs@OEG could be easily perceived via the protein denaturation at high temperatures—see Additional file 1: Figure S1.3. Irradiation of the Albumen containing 0.3 nM of AuNPs@OEG led to a dramatic increase in temperature (ΔT = 24 °C), whereas without AuNPs@OEG the temperature only increased by 4.5 °C (Additional file 1: Figure S1.3). Increasing concentrations of AuNPs@OEG led to complete coagulation of the albumen (“boiled egg”) due to the effective rise in temperature (Additional file 1: Figure S1.3 and S1.4). This clearly demonstrates the heating efficacy of AuNPs@OEG with 13 nm in diameter when irradiated at their LSPR peak, where relatively low concentrations can induce high temperature variations in biological matrices.

**Fig. 2:**
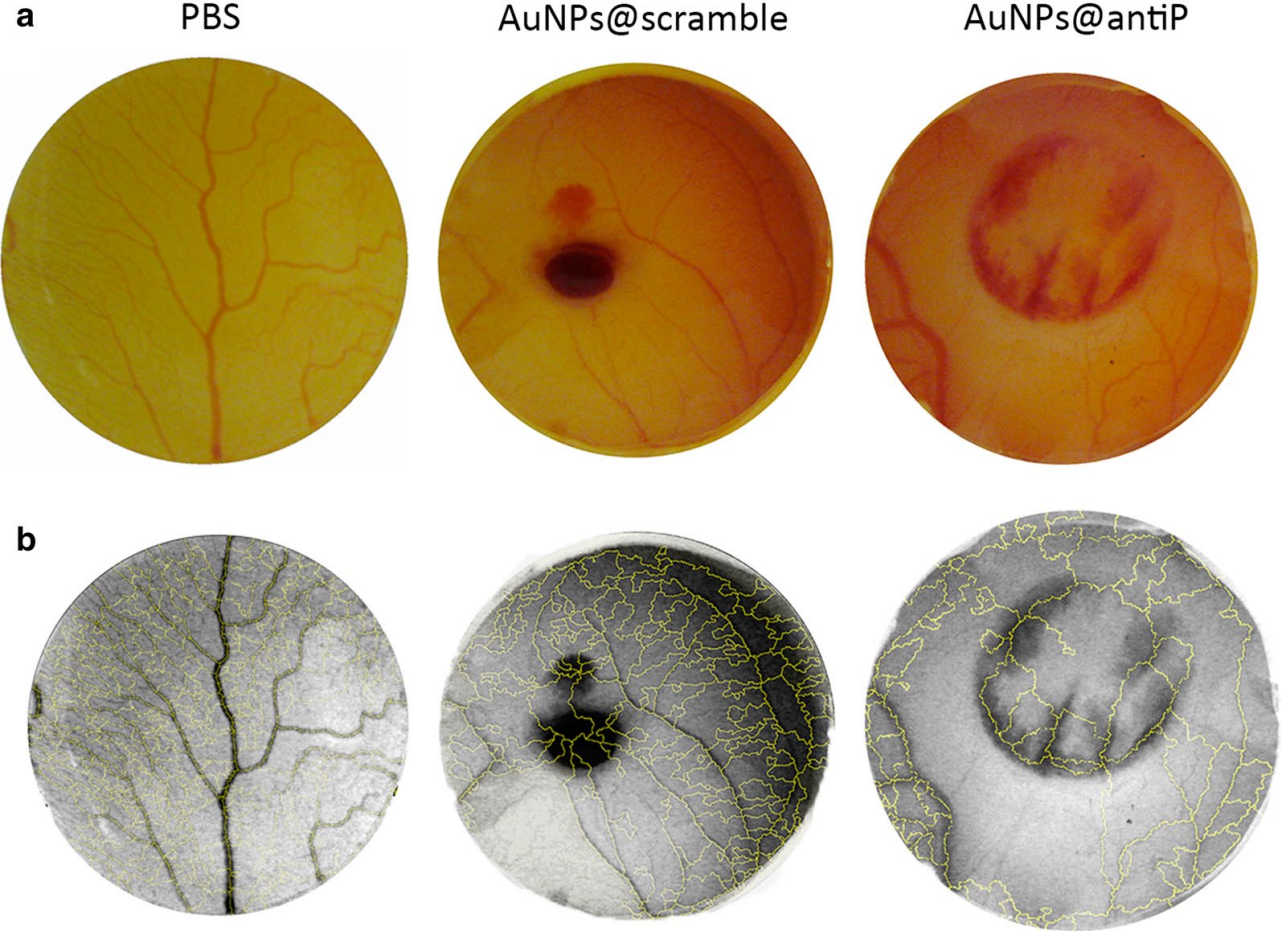
**a** Green Laser Irradiation of blood (87 μL) mixed with AuNPs@OEG (16.4 nM) or water (Vf-100 µL) at varying potencies for 1 min at of AuNPs@OEG. Cauterization of the blood samples can be seen as black spots. **b** Infrared image of the blood well with nanoparticles irradiated with 0.61 W cm^2^. The sample achieved a final temperature of 92 °C

**Fig. 3:**
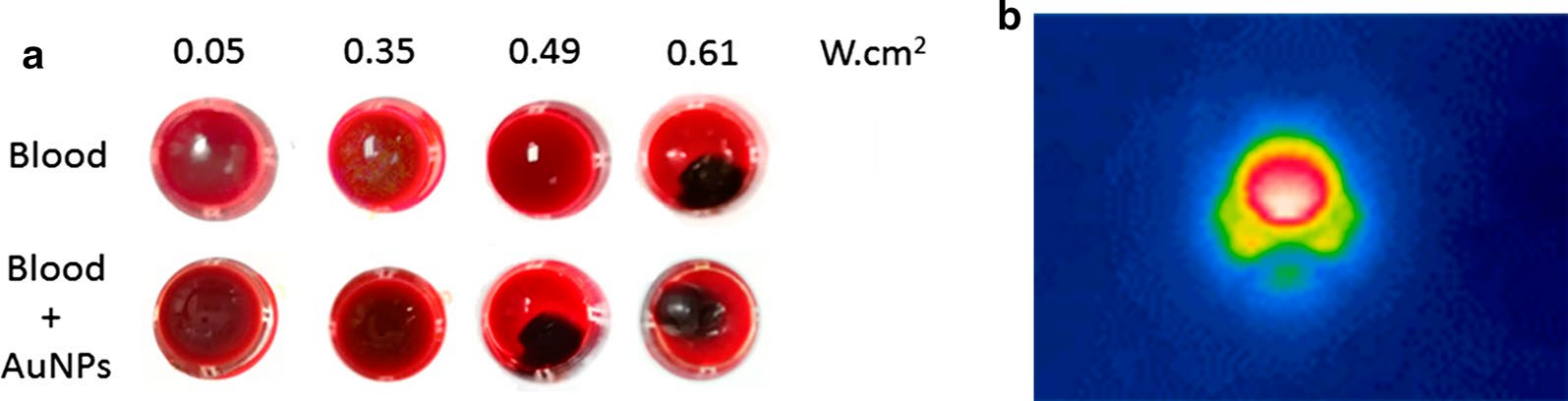
CAM images 24 h after application of the nanoformulation and irradiation. **a** Images before software analysis. **b** Images after software analysis. The nanoparticle formulations were at 16.4 nM concentration and the irradiated conditions were exposed to 0.49 W cm^−2^ for 60 s

**Fig. 4:**
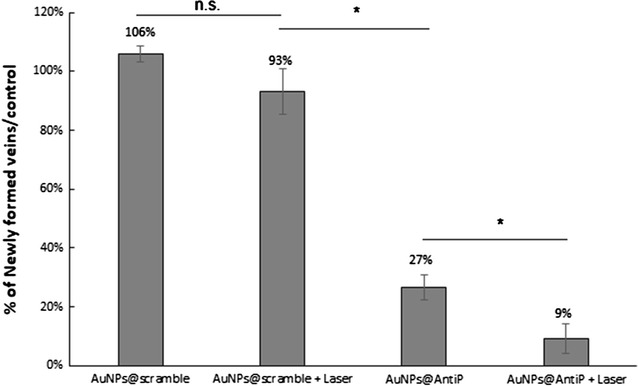
Percentage of newly formed vessels over 24 h in CAM model compared with respective control. AuNPs@scramble (16.4 nM) were compared with PBS and AuNPs@antiP (16.4 nM) were compared with AuNPs@scramble. Irradiated formulations (Laser) were exposed to 0.49 W cm^−2^ for 60 s. Differences between groups were considered significant at P < 0.05 (*)

We then assessed the effect of irradiating a blood sample with and without AuNPs@OEG, at a previously determined concentration that inhibits angiogenesis (16.4 nM), to mimic blood capillary cauterisation [13]. One minute of irradiation at 0.61 W cm^−2^ without AuNPs@OEG caused blood to coagulate, thus setting the upper threshold for irradiation. Between 0.49 and 0.61 W cm^−2^, blood only cauterises in the presence of AuNPs@OEG, determining the effective window for the CAM assay (Fig. 2). The ex-ovo CAMs were divided into two groups: one exposed to AuNPs@scramble and AuNP@antiP and kept in the dark; and the test group, where embryos were exposed to each nanoformulation and irradiated with a potency of 0.49 W.cm^−2^ for 1 min with the green laser. An additional control ring with a saline solution (phosphate-buffered saline, PBS 1×) was also used. The percentage of the newly formed arterioles was compared from images taken at 0 and 24 h, as previously described [29] (Fig. 3 and Additional file 1: Figure S1.5). A cauterisation spot, ranging from 0.8 to 2.6 mm in diameter, may be observed solely for the CAMs challenged with AuNPs and irradiated with the green laser (Fig. 3). Coagulation occurs due to photothermal conversion by AuNPs, and not by the laser alone. This shows that irradiation of AuNPs can be used for safe localised photothermal cauterisation without affecting the surrounding vessels. One can also observe that, the AuNPs@scramble alone or after irradiation do not affect the formation of new blood vessels when compared with PBS (Fig. 4). The anti-angiogenic nanoformulation (AuNPs@antiP) in the group kept in dark, induced a 73% reduction in the formation of new arterioles when compared to the scramble nanoconjugate, in accordance to previously reported results [29]. What is more, irradiation of the nanoconjugates potentiates this effect, mounting on its anti-angiogenic effect and leading to a 91% reduction of newly formed arterioles. Neuropilin-1 is a protein encoded by the *NRP*-*1* gene in humans and has been shown to interact with vascular endothelial growth factor A (VEGFA) forming a co-receptor complex with VEGFR-2 and mediating VEGF signalling [14, 15]. *VEGFA* encodes for the most common growth factor of VEGF pathway. Fms Related Tyrosine Kinase 1 (*FLT*-*1*) encodes for the human VEGFR-1. It is a transmembrane protein with tyrosine kinase activity, important for the signalling of cell proliferation and differentiation, where the main function of VEGFR-1 is to regulate blood vessel morphogenesis. NRP-1 expression is upregulated in several human tumours, e.g. brain, prostate, breast, colon, and lung cancers, promoting the vascularisation and progression of cancer, as well as contributing to invasiveness and metastasis [3, 4].

**Fig. 5:**
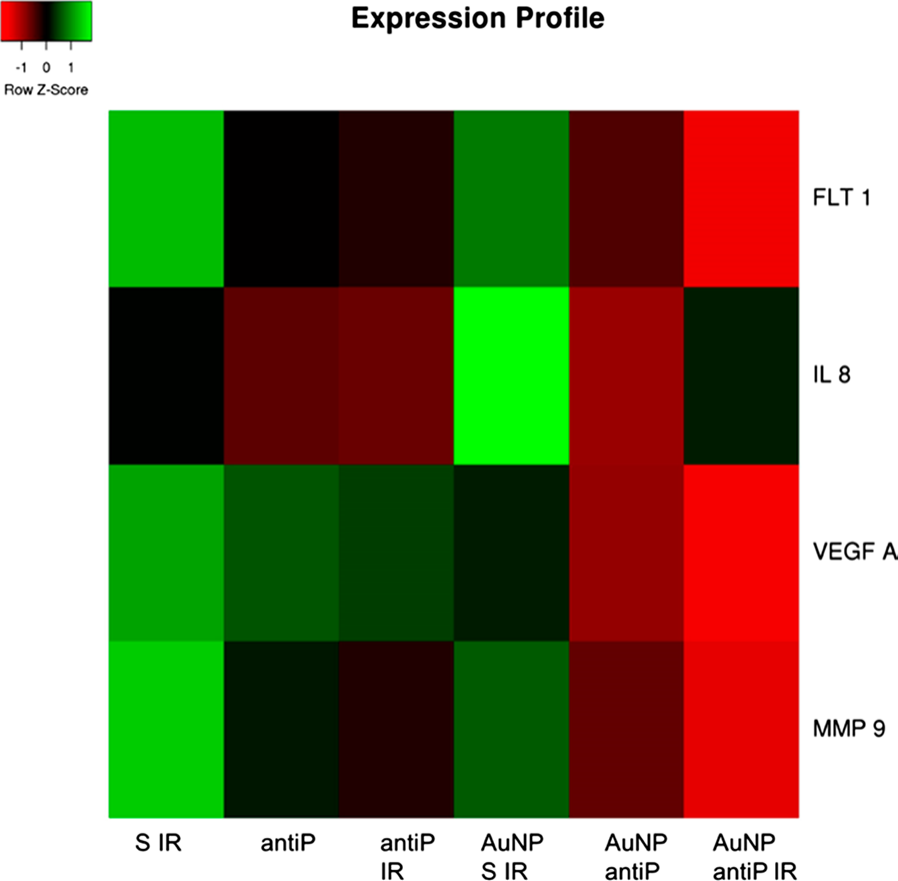
Gene expression heatmap for data retrieved from qPCR. Gene expression measured for scramble peptide irradiated (S IR); antiP and antiP irradiated (antiP and antiP IR, respectively); and the respective nanoconjugates. The nanoparticle formulations were at 16.4 nM concentration and the irradiated conditions were exposed to 0.49 W cm^−2^ for 60 s

To further understand the molecular mechanisms of angiogenesis modulation by the AuNP@antiP, we used qPCR to look for variations in mRNA expression of genes involved in VEGF pathway, interleukin 8 (*IL*-*8*), *VEGFA* and metalloproteinase 9 (*MMP*-*9*), when compared to the scramble peptide alone or conjugated to AuNPs—Fig. 5. *MMP*-*9* is a matrix metalloproteinase involved in the breakdown of extracellular matrix, and facilitates the vascularisation network growth [6, 30, 31]. Evidence show that *MMP*-*9* is up-regulated in some tumours and plays an important role in cancer angiogenesis, possibly by facilitating the access of VEGF to its receptors [6, 30, 31]. Concordantly, down-regulation of MMP-9 was shown to repress metastasis [6, 30, 31]. IL-8 is a chemokine that enhances endothelial permeability during early stages of angiogenesis, which requires activation of VEGFR-2 [32–35]. No alterations to gene expression were observed with the scramble peptide, neither free nor in nanoconjugate, when irradiated. This indicates that laser irradiation per se does not affect VEGF pathway, despite vascular cauterisation. Irradiation of AuNPs@antiP promotes a clear downregulation of *FLT*-*1* and *VEGFA*, 4- and 3.5-fold, respectively. Together, these data indicate that *NRP*-*1* regulation over *FLT*-*1* and *VEGFA* expression is clearly enhanced upon laser irradiation. Our results show that *MMP*-*9* is down-regulated in embryos exposed to AuNPs@antiP by 1.9-fold and further repressed when irradiated (down 3.5-fold). *IL*-*8* expression was not altered by the peptides or irradiation, indicating that the *NRP*-*1* pathway does not influence *IL*-*8* expression in our CAM angiogenesis model—see Additional file 1: Figure S1.7. Together, these results show that green laser irradiation of AuNPs@antiP modulates neovascularisation via VEGF pathway, regulating *FLT*-*1*, *MMP*-*9*, *VEGFA* expression but not *IL*-*8*. In vivo cauterisation of vessels coupled to blockade of angiogenesis clearly shows the potential to be used in cancer therapy, hindering vascular supply required for tumour progression.

## Conclusions

Here, we showed that spherical 13 nm gold nanoparticles conjugated to anti-angiogenic peptides may be used as efficacious photothermal agents in the visible region of the spectrum. These conjugates can denature albumin and haemoglobin via an extremely focalised irradiation that does not require guide light, since it is itself visible. This way, existing surgical green laser mays be used directly to cauterise tissues harbouring these nanoconjugates. Furthermore, green laser irradiation of the gold nanoconjugates potentiates the anti-angiogenic effect in vivo via down regulation of the VEGF pathway. With this approach, we can specifically destroy vessels surrounding the tumour by laser ablation and inhibit neovascularisation through NPR-1 blockage. The proposed combination of chemo- and phototherapy allows for a reduction of dose of anti-angiogenic peptide, thus reducing possible side effect to the neighbouring tissues, while arresting blood vessel supply critical for tumour progression, which might become a valuable strategy for cancer treatment.

## Methods

### Laser characterisation

Actinometry measurements were performed to a continuous 532 nm green diode-pumped solid-state laser (Changchun New Industries Optoelectronics Tech. Co., LTD, Changchun, China) coupled to an optical fiber, with actinometer Aberchrome 540TM, E-form ((E)-a-(2,5-dimethyl-3-furylethylidene)(isopropylidene) succinic anhydride)) (Extrasynthese, France) following literature recommendations [36]. Briefly, when the dye is exposed to UV light, due to its reversible photocyclization, it is converted into an isomer 7,7a-dihydro-2,4,7,7,7a-pentamethylbenzo(b)furan-5,6-dicarboxylic anhydride (C-form). When irradiated with visible light, it can in turn be reverted to the E-form. For these measurements, a solution of 100 µM of Aberchrome 540 was dissolved in absolute ethanol and irradiated at 342 nm for 1 h (h) until a photo-stationary state corresponding to the maximum conversion into the C-form was obtained. The C-form solution was irradiated using a continuous 532 nm green diode-pumped solid-state laser (Changchun New Industries Optoelectronics Tech. Co., LTD, Changchun, China) coupled to an optical fibre. The solution exposed to the green laser undergoes back conversion to the E-form and the converted molecules are quantified by UV–Vis spectroscopy (Shimadzu). For the calculation of the photon flux, a photochemical quantum yield of ϕ = 0.060 was considered (data not shown). To characterise the effect of the laser and the AuNPs@OEG in the embryos, Albumen and blood were separately disposed in multi-well plates. Initial temperature was measured with a thermocouple (Digital Multimeter, MY-64, MASTECH, Hong Kong). After irradiation, the final temperature was measured and images were taken. The same procedure was repeated with AuNPs@OEG mixed with each component.

### Synthesis and functionalisation of AuNPs

Spherical gold nanoparticles (13 ± 2 nm) were synthesised following published literature procedures [18, 28]. Briefly, to a boiling aqueous solution of sodium tetrachloroaurate (100 mL, 1 mM) was added a hot aqueous solution of trisodium citrate (5 mL, 2% wt/V) under rapid stirring. Once a deep red colour, indicating the formation of nanoparticles, was observed, the solution was continuously stirred for 15 min. Afterwards the stirring solution was allowed to cool to room temperature. As prepared citrate-capped AuNPs were sterile-filtered using a 0.2 µm syringe filter and stored at 4 °C until further use. For functionalisation, a freshly prepared aqueous solution of monocarboxy (1-mercaptoundec-11-yl) hexaethylene glycol (OEG, MW = 526.7 g mol^−1^, 200 μL) was added to a moderately stirring gold nanoparticle solution (10 mL, 5 nM). The mixture was continuously stirred for 2 h followed by an overnight incubation at 4 °C. AuNPs@OEG were then purified from excess OEG by three rounds of centrifugation (16,400 rpm, 15 min) and re-dispersion in borate buffer (10 mL, 0.01 M, pH 9). AuNPs@OEG were stored at 4 °C until further use.

Scramble and antiP peptides were then conjugated to AuNPs@OEG as previously reported [18, 37]. The peptide solution (100 μL, 1 mg mL^−1^, MW scramble = 922.1 g mol^−1^, MW antiP = 968.2 g mol^−1^, in 0.01 M sodium borate buffer, pH 9) was added to AuNPs@OEG (5 mL, 1.5 nM in 0.01 M sodium borate buffer, pH 9) followed by aqueous solutions of EDC (1-(3-(dimethylamino)propyl)-3-ethyl-carbodiimidemethiodide, 50 μL, 0.2 M) and sulfo-NHS (N-hydroxysulfosuccinimide, 100 μL, 0.2 M). After shaking at room temperature for 24 h, the reaction mixture was purified by three rounds of centrifugation (16,400 rpm, 15 min) and re-dispersion in water followed by lyophilisation.

### Ex ovo CAM assay

All the in vivo CAM experiments were performed according to the Directive 2010/63/EU of the European Parliament and of the council of 22 September 2010 on the protection of animals used for scientific purposes, which does not contain any kind of restriction to the use of non-mammal embryos. The use of this alternative animal model fits in the “3Rs policy” that was strictly followed during experiments.

Fertilised eggs were incubated at 37 °C, 99% (v/v) relative humidity, until 72 h and gently opened into a petri dish, with the chicken chorioallantoic membrane (CAM) facing upwards. Afterwards four transparent plastic, O-rings (6 mm inside and 9 mm outside diameter) were placed on the CAM. Inside each O-ring, 40 μL of free peptides or peptide-conjugated AuNPs were pipetted (0.01 pmol μL^−1^ for peptide concentration) [29].

For blood vessel modulation experiments each O-Ring per CAM contained PBS, scramble and antiP in the free form or in formulation with AuNPs (AuNPs@scramble and AuNPs@antiP), and were further irradiated or kept in dark. Thus, each CAM is exposed to four different stimuli, one in each O-ring. After stimuli, the embryos were then incubated at 37 °C, 99% (v/v) relative humidity for 24 h. Images were acquired at 0 and 24 h of incubation using a Digital USB Microscope Camera (Opti-Tekscope OT-V1).

For mRNA expression analysis, all the O-rings in each CAM were pipetted with the same solution of PBS, scramble and antiP, in free form or in formulation with AuNPs, and were after irradiated or kept in dark. Thus, each CAM was exposed to only one stimuli, the same in all O-rings. After stimuli, the embryos were then incubated at 37 °C, 99% (v/v) relative humidity for 24 h and total mRNA was extracted.

### Blood vessel modulation experiments

After image acquisition, the internal area of each O-ring was extracted from all imagens, then the green channel of the images was extracted using ImageJ 1.49k (Wayne Rasband, National Institutes of Health, USA). For image analysis, a noise tolerance of eight was considered for segmentation, excluding the edge maxima and binary performed. Using the skeleton plugin of ImageJ the number of branches was counted within the region treated with the sample formulations. The blood vessels in the image obtained after channel split were also counted manually for comparison. The formed blood vessels were calculated for 0 and 24 h. The percentage of newly formed vessels is given by dividing the values at 24 h by the values at 0 h.

### RNA expression analysis

Total RNA was extracted from CAM using TRIsure™ Reagent method (Bioline, London, UK) according to the manufacturer’s instructions. The quality of RNA was assessed using the Nanodrop ND-1000 Spectrophotometer (ThermoScientific, Wilmington, DE). Total RNA extracted from samples was reverse transcribed using the NZY M-MuLV First-Strand cDNA Synthesis kit (Nzytech, Lisbon, PT) followed by qPCR amplification using KAPA SYBR^®^ FAST kit (KAPAbiosystems, Cape Town, SA). The following conditions were used: initial denaturation at 95 °C for 5 min; 40 cycles of 95 °C for 30 s, Tm  °C for 30 s, 72 °C for 1 min; and a final extension step at 72 °C for 10 min. The Tm temperature varied from set of primers, as described in Additional file 1: Table S1.5.

### Statistical analysis

Results are presented as average ± standard deviation of triplicate experiments. An unpaired, parametric, T-student test was performed between each group and the respective scramble. Differences between groups were considered significant at P < 0.05 using GraphPad Prism 7.00.

## Additional file

**Additional file 1.** Additional tables and figures.

## Abbreviations

VEGF: vascular endothelial growth factor
EPR: enhanced permeability and retention
AuNPs: gold nanoparticles
NRP-1: neuropilin-1 receptor
HUVEC: human umbilical vein endothelial cells
LSPR: localised surface plasmon resonance
CAM: chorioallantoic membrane
OEG: oligo ethylene glycol
VEGFA: vascular endothelial growth factor A
FLT-1: Fms Related Tyrosine Kinase 1
IL-8: interleukin 8
MMP-9: metalloproteinase 9
