# Supplementary material for: Potentiating angiogenesis arrest in vivo via laser irradiation of peptide functionalised gold nanoparticles
Source: J Nanobiotechnology. 2017 Nov 21;15:85. doi: 10.1186/s12951-017-0321-2 (PMC5697398; doi:10.1186/s12951-017-0321-2)
Supplement: Supplementary file 1 — Additional file 1. Additional tables and figures. [file 12951_2017_321_MOESM1_ESM.docx]

**Additional File 1**

**Potentiating angiogenesis arrest *in vivo* via laser irradiation of peptide functionalised gold nanoparticles**

Pedro Pedrosa^a^, Amelie Heuer-Jungemann^b^, Antonios G. Kanaras^b^, Alexandra R. Fernandes^a^ and Pedro V. Baptista^a^,*

^a^UCIBIO, Departamento de Ciências da Vida, Faculdade de Ciências e Tecnologia, Universidade NOVA de Lisboa, Caparica, Portugal

^b^Institute for Life Sciences, Physics and Astronomy, Faculty of Physical Sciences and Engineering, University of Southampton, SO17 1BJ, U.K.

*Email: pmvb@fct.unl.pt

##

## S1.1 Nanoconjugate characterisation

**Table S1.1-** Peptide Amino Acid Sequences

| **Name** | **Function** | **Sequence** |
| --- | --- | --- |
| **Scramble** | Scramble control | KPRQPSLP |
| **AntiP** | Anti-angiogenic | KATWLPPR |

**
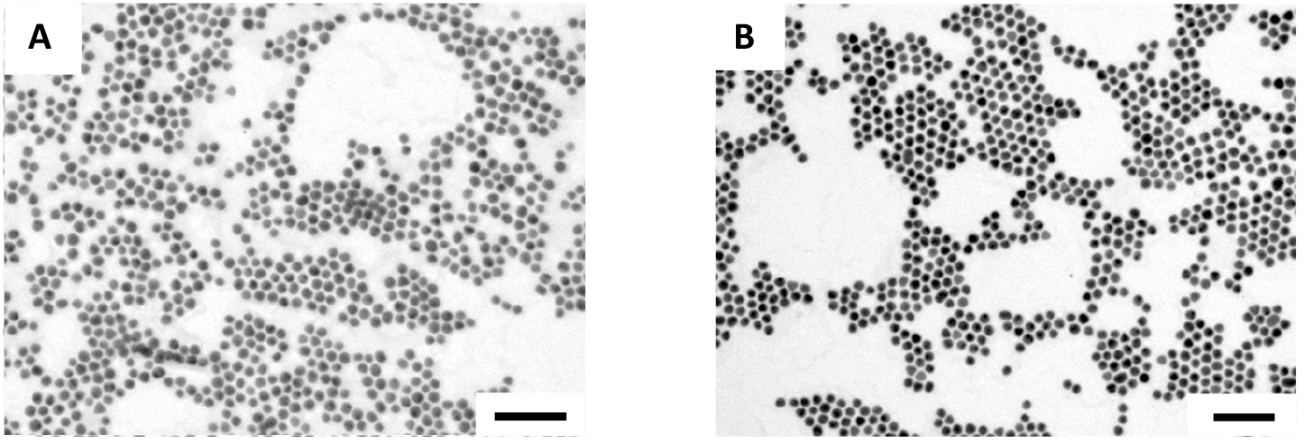
**

Figure S1.1- TEM images of AuNPs@antiP **A)** and AuNPs@scamble **B)**. Scale bars - 100nm.

AuNPs@OEG show an average size of 21.0 ± 0.2 nm by DLS. Zeta potential measurements of AuNPs@OEG showed a negative charge (-27.53 ±1.5 mV) possible due to the presence of the carboxylic groups. AuNPs@scramble show a hydrodynamic diameter of 22.7 ± 3.3 nm while zeta potential changed by 4 mV (-23.40 ±2.3 mV) due to the formation of amide bonds between the carboxylic groups of the outer shell of OEG and the N-terminus lysine’s in the positively charged peptides; AuNPs@antiP had a slightly lower net charge with an average size of 22.2 ± 2.2 nm. All measurements were obtained in PBS 1X.

## S1.2 Laser Characterisation

Figure S1.2- a) Linear relation between input laser current and light power arriving to the embryos. b) Heat generated per nM of AuNPs in Albumen matrix after irradiation with 3.83 W.cm^-2^ during 2 min.

## S1.3 Laser effect on albumen


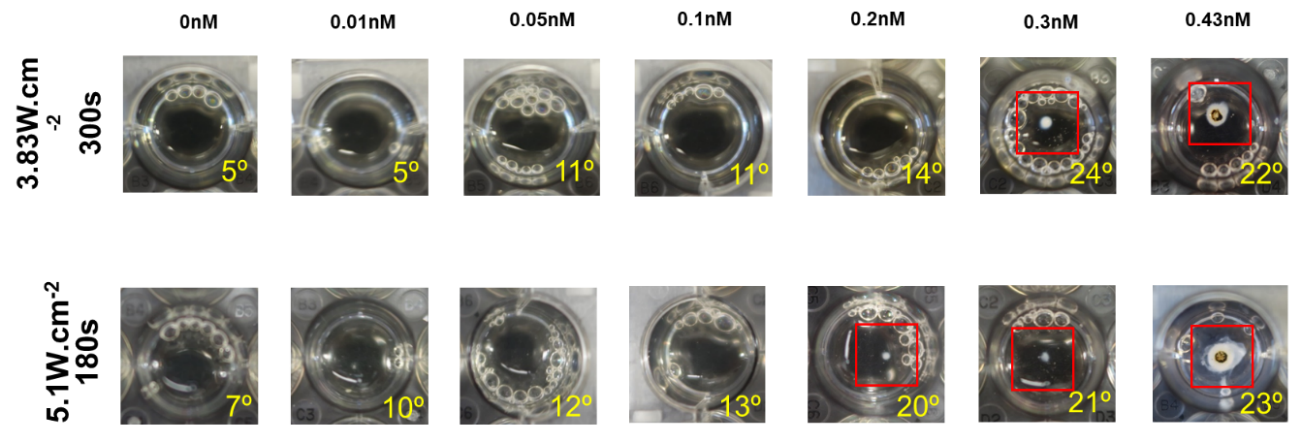


Figure S2.3- Green laser irradiation of albumen (500uL) mixed with AuNPs@OEG (Vf- 700uL) at fixed potencies and time periods, varying gold nanoparticle final concentration. The ∆T (ºC) after irradiation is represented at the bottom left of each assay. The initial temperature was 29 °C ± 1 °C for all assays.

## S1.4 Temperature variation on Albumen

Figure. S2.4 Temperature variation of albumen after green laser irradiation of albumen (500uL) mixed with AuNPs@OEG (Vf- 700 µL) at fixed potencies and time periods varying gold nanoparticle concentration. The initial temperature was 29 °C ±1 °C for all assays.

## S1.5 CAM Images

**b)**

**a)**


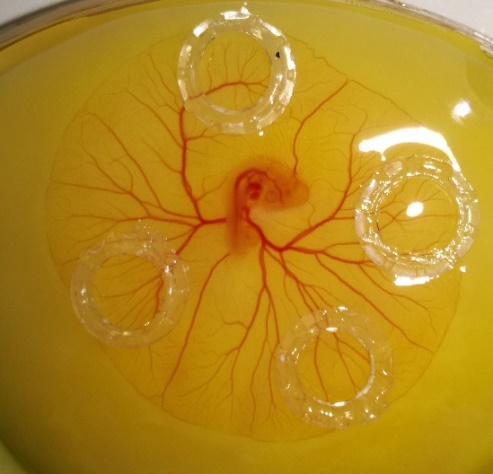

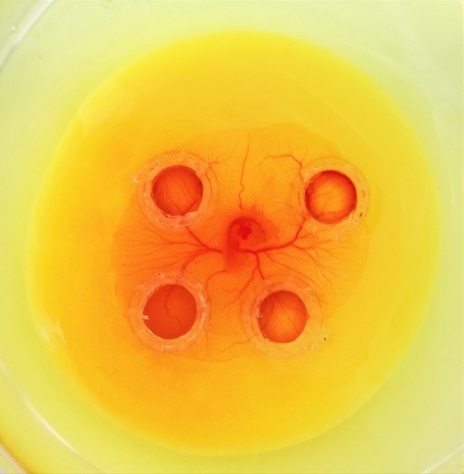


Figure S1.5- Photographs of CAM a) before application of AuNPs@antiP and b) after application of AuNPs@antiP at 16.4 nM.

**S1.6 Primers Table**

**Table S1.6**- List of primers used for gene expression analysis

| Primers | Sequence 5'-3' | Size (bp) | Tm (°C) |  |
| --- | --- | --- | --- | --- |
| GAPDH FW | GAGGAAAGGTCGCCTGGTGGATCG | 290 | 55 |  |
| GAPDH RV | GTGAGGACAAGCAGTGAGGAACG |  |  |  |
| IL-8 FW | TCACAGCTCCACAAAACCTCA | 120 | 60 |  |
| IL-8 RV | CGTCCTACCTTGCGACAGA |  |  |  |
| VEGFR-A2 FW | AGAAAATCACTGTGAGCCTTGCT | 101 | 60 |  |
| VEGFR-A2 RV | TGCAACGTGAGTCTGTGAATTTG |  |  |  |
| FLT-1 FW | TCGACACTATCTTCACAGCGG | 95 | 60 |  |
| FLT-1 RV | GCTTCTGCAGTTTGGGCT |  |  |  |
| MMP-9 FW | GGGGTTTCCTCACCTTCGAG | 349 | 55 |  |
| MMP-9 RV | CGGGTCTTCACCTCTAAGCC |  |  |  |

## S1.7 Gene Expression Analysis


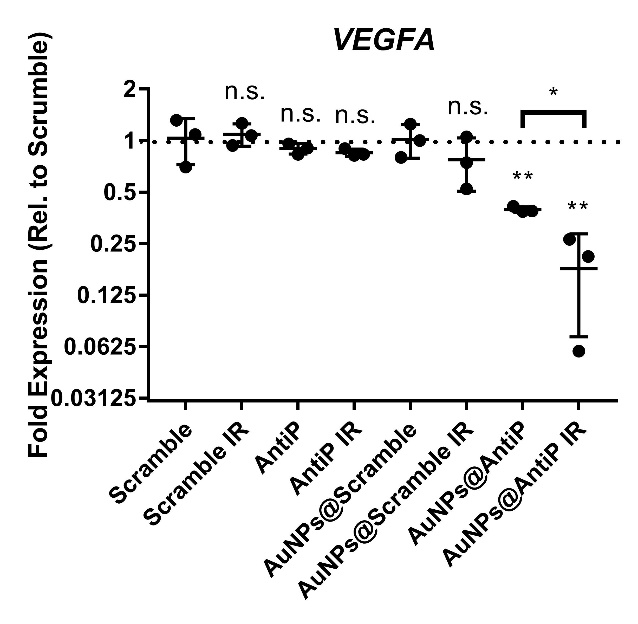

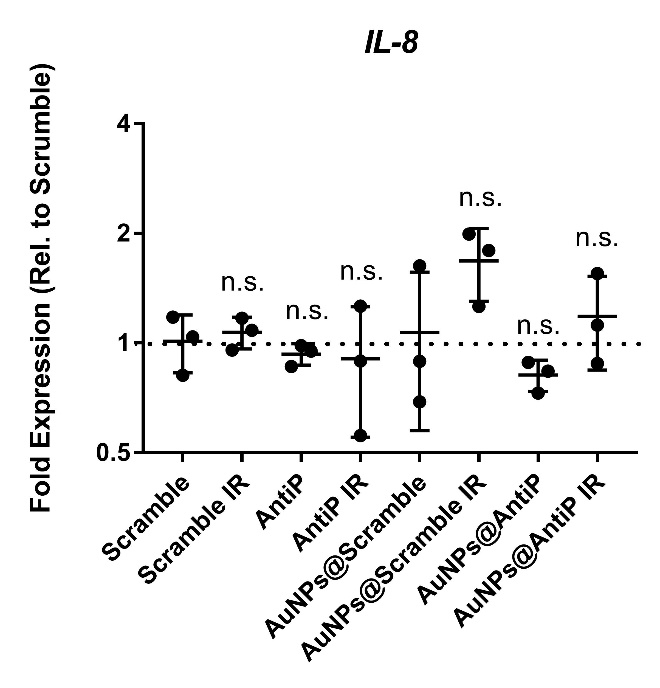

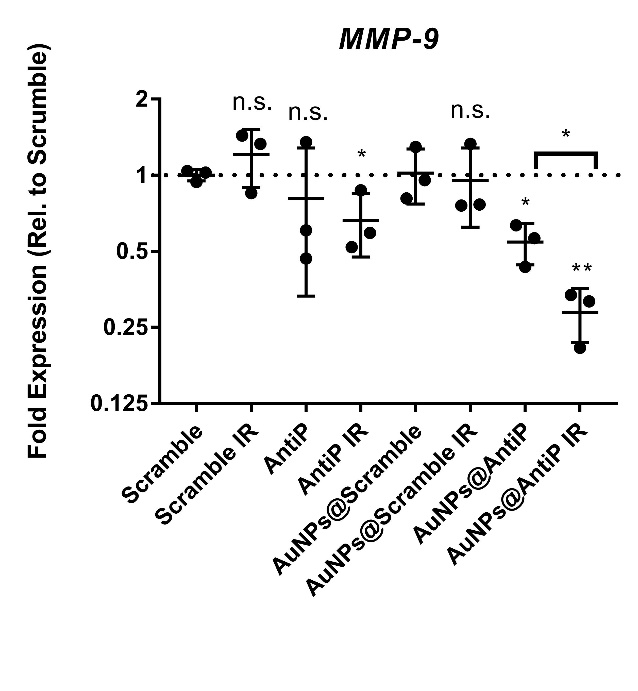

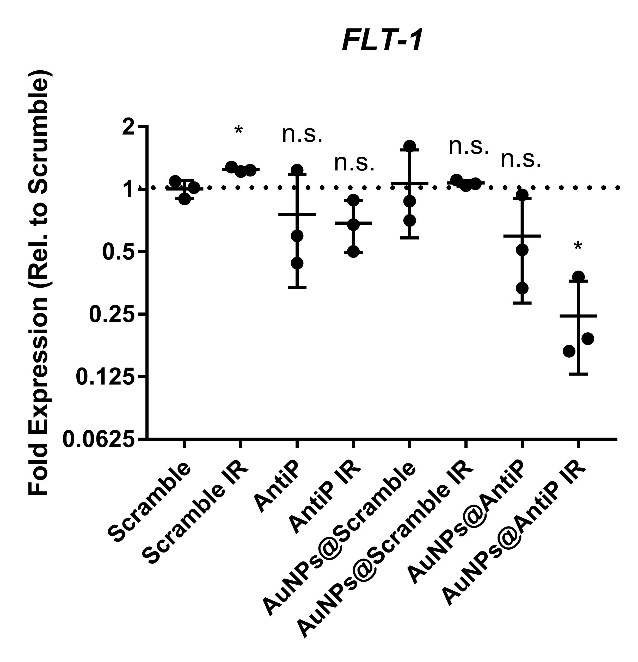


Figure S1.7- Gene expression analysis of CAM for *FLT-1*, *MMP-9*, *IL-8*, *VEGFA* genes. Normalisation was performed with housekeeping *GAPDH*. The nanoparticle formulations were at 16.4 nM concentration and the irradiated conditions were exposed to 0.49 W.cm^-2^ for 60 sec.
